# DNA5mC Regulator-Mediated Molecular Clusters and Tumor Microenvironment Signatures in Glioblastoma

**DOI:** 10.3389/fcell.2022.1055567

**Published:** 2022-11-08

**Authors:** Xinyu Yu, Yijun Che, Weiyang Li, Peng Zhang, Yunhu Yu, Jialin Chen, Ting Gong, Fang Cao

**Affiliations:** ^1^ Department of Neurosurgery, Affiliated Hospital of Zunyi Medical University, Zunyi, China; ^2^ Clinical Research Center for Neurological Disease, The People’s Hospital of HongHuaGang District of ZunYi, Zunyi, China; ^3^ Department of Neurosurgery, The Third Affiliated Hospital of Zunyi Medical University, Zunyi, China; ^4^ Department of Neonatology, The Third Affiliated Hospital of Zunyi Medical University, Zunyi, China; ^5^ Department of Quantitative Health Sciences, John A. Burns School of Medicine, University of Hawaii at Manoa, Honolulu, HI, United States

**Keywords:** DNA5mC, signatures, molecular clusters, tumor microenvironment, glioblastoma

## Abstract

Growing evidence links DNA methylation to tumor immunity. The impact of DNA methylation (5 mC) on the microenvironment surrounding tumors and immunotherapy remains to be clarified. Through clustering gene expression of 20 DNA methylation regulators, this study aimed at systematically analyzing DNA methylation regulator patterns and tumor microenvironment characteristics of TCGA-GBM patients. Various subtypes of glioblastoma exhibit different tumor microenvironments and DNA methylation patterns. Each DNA methylation modification was then assigned a DNA methylation score (DMS). High DMS was associated with a good prognosis. In contrast, the low DMS group had a relatively low survival rate. A correlation was also found between high DMS and enhanced immunotherapy efficacy in two immune checkpoint blocking treatment cohorts. To conclude, identifying DNA methylation regulation patterns may prove critical to understanding glioblastoma progression and differentiation, as well as future therapeutic targets.

## Introduction

A critical epigenetic mechanism in tumor development and progression is DNA methyltransferase-mediated methylation of cytosine to produce 5-methylcytosine (5 mC) (Ghigolea et al., 2015; Angeloni and Bogdanovic, 2019). DNA methylation has been identified as one of the factors regulating chromatin structure, conformation, stability, and protein interactions, which in turn affects gene expression (Miranda and Jones, 2007; Chen et al., 2022; Gong et al., 2022; Liu et al., 2022; Mattei et al., 2022). By binding proteins to methylcytosine, methylated DNA suppresses gene expression *via* inhibiting transcription factor binding (Moore et al., 2013).

There are approximately 7,000 cases of glioblastoma multiforme (GBM) every year (Zheng et al., 2022). A patient with GBM has a poor prognosis after treatment (Smoll et al., 2013; Alifieris and Trafalis, 2015), with a median survival rate of only 15 months (median survival). In addition to its genomic and transcriptome dimensions (Sottoriva et al., 2013; Lee et al., 2017; Cao et al., 2021a; Zhong et al., 2021), glioblastoma tumor heterogeneity appears to contribute to therapeutic resistance and relapse (Klughammer et al., 2018). A considerable amount of research is needed to clarify the role of the epigenome in the progression of glioblastoma disease. It has been shown in accumulating evidence that GBM is associated with epigenetic alterations, such as hypermethylation of tumor suppressor genes. For patients with malignant gliomas who respond to alkylating drugs, Esteller and colleagues identified a correlation between MGMT hypermethylation and an improved prognosis (Esteller et al., 2000). Several CpG islands were methylated at multiple loci in IDH1/2mut glioblastomas (Noushmehr et al., 2010). FZD9, TNFRSF10A, MEST, TES, PRKCDBP, CD81, and HOXA11 have also been identified as targets of methylation in GBM (Martinez et al., 2009).

A combination of checkpoint blockade-based immunotherapy with traditional surgery, radiotherapy, and chemotherapy has been shown to improve survival in patients with GBM (Urbańska et al., 2014). Patients with GBM can experience improved short-term survival when they receive a whole-cell lysate dendritic cell vaccine as adjuvant immunotherapy (Cho et al., 2012). A variety of biomarkers have been identified as potential biomarkers for response to PD-1 blockade-based immunotherapy, including PD-L1 expression, microsatellite instability, deficient mismatch repair, and tumor mutation burden (Davar et al., 2015; Dudley et al., 2016; Chen et al., 2018; Wang et al., 2019). PD-1/PD-L1 blockade can be used as a new biomarker to assess T cell rejuvenation associated with exhaustion (Ghoneim et al., 2017). A recently published report shows that CD96 mutation can be used as a biomarker for immune checkpoint blocking therapy in GBM (Zhang et al., 2020a). This study investigated the expression of 20 genes that regulate DNA methylation in GBM to better understand how they affect the immune microenvironment and immunotherapy efficacy. An unsupervised clustering technique identified three distinct DNA methylation regulatory patterns with differing characteristics of the immune microenvironment. To assess DNA methylation status individually, a DNA methylation score (DMS) system was developed. According to our findings, DMS may be a useful biomarker for predicting immunotherapy effectiveness.

## Materials and methods

### Collection of glioblastoma multiforme expression profile and clinical data

As a first step, we use the TCGA database (https://portal.gdc.cancer.gov/) to download data related to GBM expression profiles and clinical follow-up information. Following are the steps for processing TCGA-GBM RNA-Seq data: (Angeloni and Bogdanovic, 2019) remove samples that do not have clinical follow-up information; (Ghigolea et al., 2015) remove samples that have a survival time that is unknown, less than 30 days, and no survival status; (Mattei et al., 2022) remove probes that correspond to multiple genes; (Gong et al., 2022) take the median expression from gene symbols with multiple expressions. The summary of the clinical statistics of the 143 tumor samples from the preprocessed TCGA-GBM data in Supplementary Table S1.

### Clustering of DNA5mC-related genes and identification of differentially expressed genes

Unsupervised clustering techniques were used to find DNA methylation patterns that would be effective for grouping patients for further research. From eight integrated GEO datasets or the ACRG cohort, 20 DNA 5 mC regulators were selected to study DNA modification patterns mediated (Zhang et al., 2020b). A consensus clustering algorithm was performed using the Pam method in ‘ConsensuClusterPlus’ R package (Monti et al., 2003), which repeated 1,000 times to ensure the stability of the classification.

As a result of consistently clustering based on the expression of DNA 5 mC-related genes, the tumor samples were divided into DNA 5mC-1, DNA 5mC-2, and DNA 5mC-3 subgroups. Using the ‘limma’ R package (Ritchie et al., 2015), we identified differentially expressed genes (DEGs). We have described the data collection process and analysis elsewhere (Cao et al., 2020; Cao et al., 2021b; Mao et al., 2021). Using the Ensemble genome annotation files, we extracted functional annotations in DEGs based on a significance threshold of 0.05 and log2 (fold change) > 1.

### Gene features dimensionality reduction and construction of DNA methylation score model

DNA5mC-related DEGs were used to construct a DNA methylation score (DMS) model of tumors. To reduce noise or redundant genes, we first performed a univariate Cox regression analysis for each DEG. Z-scores were calculated based on the expression of these DEGs with significant prognosis. A DMS score was calculated based on the principal component analysis (PCA). Both PC1 and PC2 were chosen as signature scores. Using this strategy, the score was concentrated on the set with the greatest block of highly correlated genes by down-weighting contributions from genes that did not track with other members of the set. As shown in the following formula, i represents the expression of DNA5mC regulator related genes (Sotiriou et al., 2006; Zeng et al., 2019).

DMS=∑PC1(i)+∑PC2 (1)

### Gene set enrichment analysis and functional enrichment analysis

The Effects of Coordinated Gene Set Enrichment on Phenotypes can be evaluated using GSEA (Gene Set Enrichment Analysis). To compare differences between DNA modification patterns, we downloaded all hallmark gene sets from the Molecular Signature Database (MSigDB). We performed GO and KEGG enrichment analysis by the ‘clusterProfiler’ R package with a cutoff of *p* < 0.05 (Mao et al., 2020; Wu et al., 2021). According to fold change, DEGs were estimated and sorted between groups with high and low gene expression.

### Statistical analysis and hypothesis testing

To determine the normality of the variables, the Shapiro-Wilk test was performed. Unpaired t tests were used for statistical analysis of comparisons between two normally distributed groups, and Wilcoxon rank-sum tests were used for statistical analysis of nonnormally distributed data. Kruskal–Wallis tests or one-way ANOVA were employed as nonparametric or parametric procedures, respectively, to compare the three groups. Spearman and distance correlation analyses were used to calculate the correlation coefficients. Utilizing the survcutpoint function from the ‘survminer’ R package, the optimal cutoff values for each cohort were determined. (Kassambara et al., 2017). The Kaplan-Meier method was used to create the survival curves for the prognostic analysis, and log-rank tests were applied to see whether there were any differences between groups (Zhou et al., 2019). For DNA regulators and genes associated with DNA methylation regulator patterns, univariate Cox regression analysis was performed. A two-sided *p*-value of 0.05 was used for statistical significance. R 3.6.1 was used to perform all statistical analyses.

## Results

### Molecular characterization of DNA 5 mC mediators in glioblastoma multiforme

**FIGURE 1:**
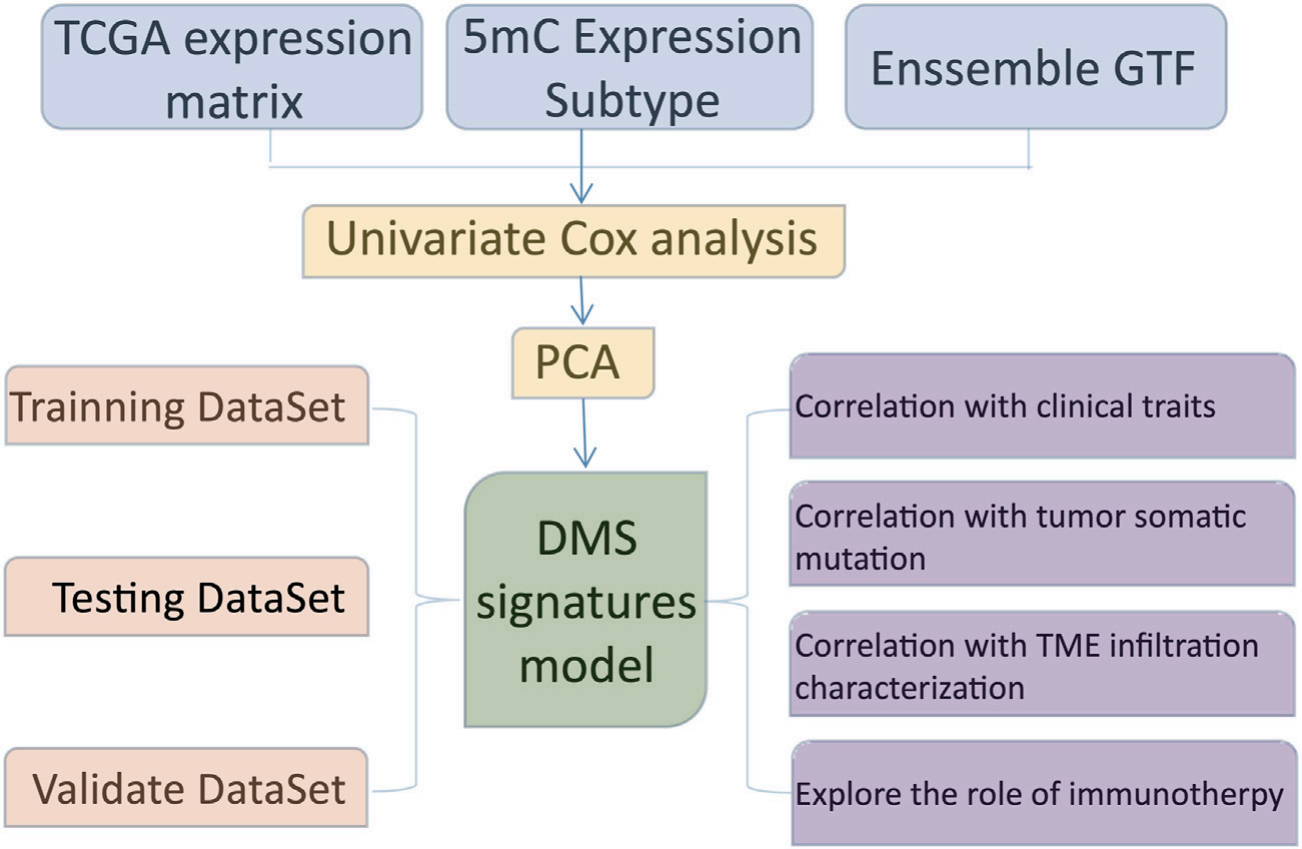
The overall design of this study.

**FIGURE 2:**
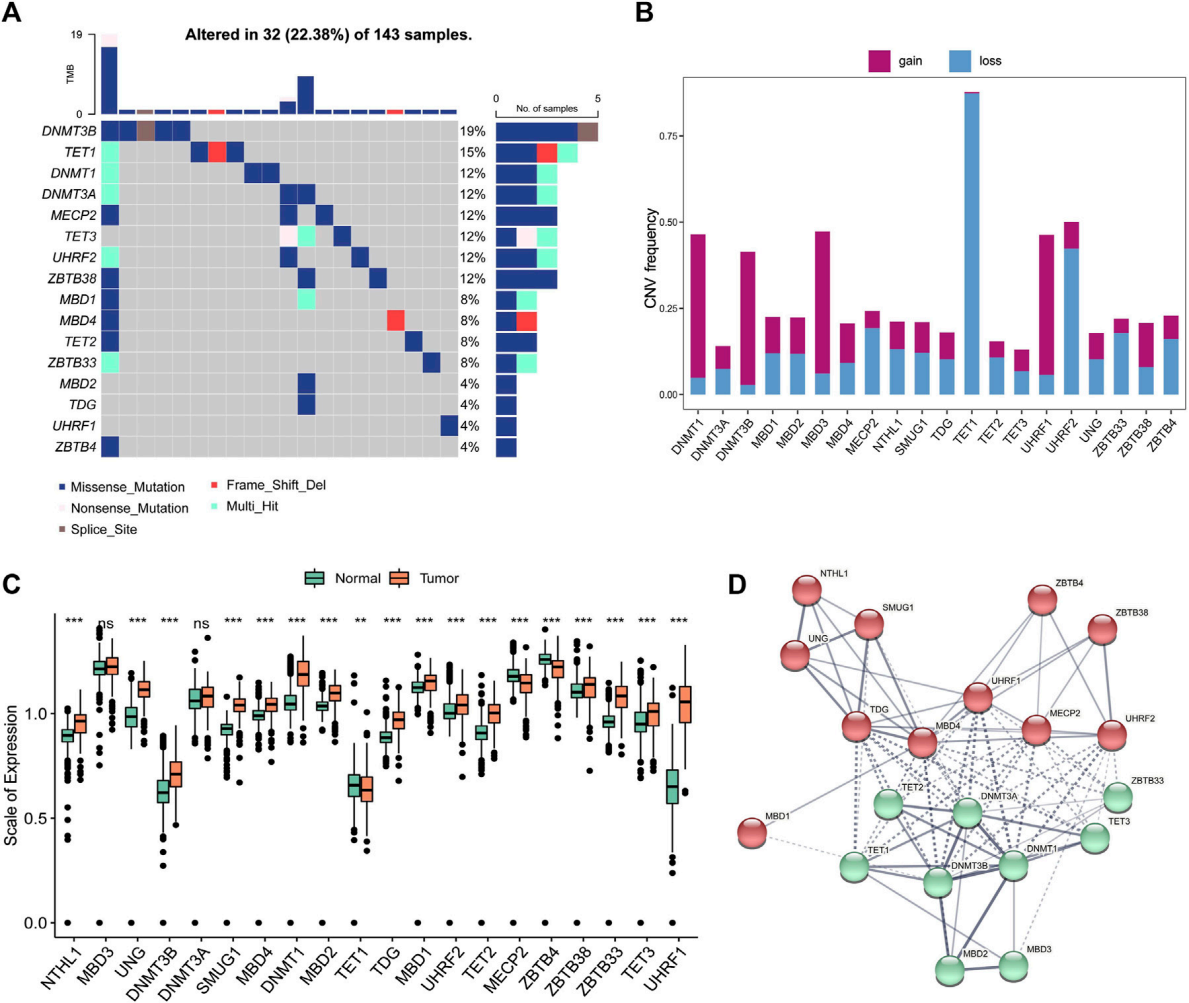
Genetic and expression variation of DNA5mC related genes in GBM **(A)** Mutation frequencies at 20 DNA5mC regulators in the TCGA-GBM cohort. **(B)** DNA5mC regulator CNV variation frequency. **(C)** A comparison of the expression of 20 DNA5mC regulators between normal tissues and GBM tissues. **(D)** Diagram of gene interaction at the protein level.

The entire study design was illustrated in Figure 1. In this study, we collected and enrolled 20 DNA methylation regulators, including 14 readers (ZBTB33, ZBTB38, ZBTB4, MBD1, MBD2, MBD3, MBD4, MECP2, UNG, TDG, NTHL1, and SMUG1), 3 writers (DNMT1, DNMT3A, and DNMT3B), and 3 erasers (TET1, TET2, and TET3). Among them, there are 16 of 20 regulators with mutation rates more than 3%, ranging from 4% to 19% (Figure 2A). The mutation rates in GBM patients were extremely low for two readers, UHRF1 and ZBTB4, while they were high for DNMT3B (19%), TET1 (15%), and DNMT1 (12%). Subsequently, 20 regulators were examined for their frequency of copy number variation (CNV) alteration. Over 50% of the TET1 gene had CNV alterations, which was prevalent across 20 regulators. Many were concerned with copy number deletions, whereas DNMT1, MBD3, UHRF1, and DNMT3B showed widespread copy number amplifications (Figure 2B).

To ascertain whether the above genetic variations influenced the expression of DNA5mC regulators, the mRNA expression levels of regulators were investigated between normal and GBM samples. The results displayed the A significant factor responsible for perturbing DNA5mC regulator expression was alterations in CNV. Several DNA5mC regulators were found to be significantly overexpressed in tumor tissues, including DNMT1, DNMT3B, TET2, TET3, MBD1, and SMUG1. In contrast to normal tissues, TET1, MECP2, and ZBTB4 with deleted CNV were prominently under-expressed (Figure 2C). A striking difference in genes and expression patterns was observed in DNA5mC regulators between normal and GBM samples, indicating that DNA5mC regulator imbalance contributes to the formation and progression of GBM. According to the STRING database (https://www.string-db.org/), DNA5mC regulators also interact with one another at the protein regulatory level (Figure 2D).

### Correlation between immune cell infiltration in glioblastoma multiforme and DNA 5 mC-related genes

**FIGURE 3:**
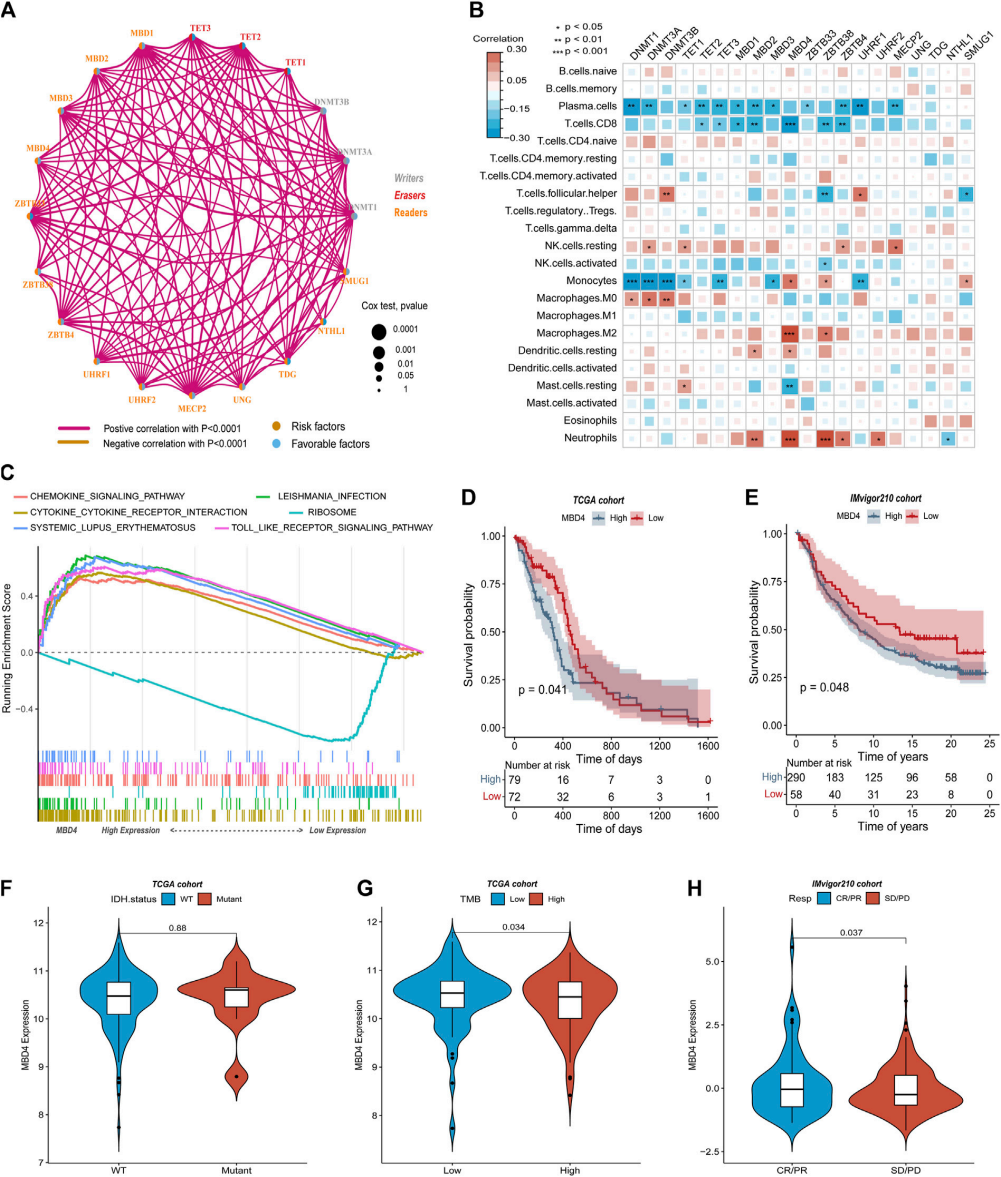
TCGA-GBM data on immune cell infiltration and DNA5mC regulators. **(A)** Coexpression network of DNA5mC regulators. **(B)** Heatmap of gene correlation with immune cell infiltration. **(C)** GSEA for high and low gene expression. **(D–E)** Survival analysis of the TCGA-GBM and IMvigor cohorts. **(F–G)** Comparison of gene expression in TCGA-GBM and IMvigor cohorts. **(H)** Gene expression differences between immunotherapy responders and non-responders.

DNA5mC genes and the tumor immune microenvironment were explored using the CIBOERSORT algorithm (Supplementary Table S2) to measure the overall infiltration of 22 immune cells, including B cells and natural killer cells. TCGA-GBM dataset was analyzed first for co-expression of DNA5mC-related genes. Most regulators showed significant positive correlations (Figure 3A). We then examined the relationship between DNA 5 mC regulator expression profiles and 22 immune cell infiltrations (Figure 3B). A distinct difference was found between different genes and immune cell infiltration. Infiltrations of Tcells. CD8, macrophages. M2, and neutrophils were strongly correlated with MBD4. To determine the effect of high and low expression of MBD4, a GSEA enrichment analysis was performed. The findings manifested a marked enrichment of immune activation pathways in samples with high gene expression levels, including the chemokine signaling pathway, cytokine-cytokine receptor interaction, and Toll-like receptor signaling pathway. On the other hand, samples with low gene expression exhibited an increase in ribosome pathways (Figure 3C).

A relationship was then investigated between MBD4 gene expression, IDH mutation status, and tumor mutation burden (TMB) in TCGA-GBM. The expression of MBD4 gene was not significantly different between groups with or without IDH mutation (Figure 3F), but the expression of MBD4 gene in high TMB groups was prominently lower than that in low TMB groups (Figure 3G). TCGA-GBM samples were further divided into two groups based on MBD4 expression levels using the optimal density gradient. There was a significant difference between the survival curves of the two groups, and the OS of the low-expression group was superior to that of the high-expression group (Figure 3D). A similar result was observed in the IMvigor210 dataset (http://researchpub.gene.com/IMvigor210CoreBiologies) (Figure 3E). Further, MBD4 was expressed more frequently in tumor immune microenvironment and tumor immunotherapy response groups than in non-response groups (Figure 3H).

### Identification and functional enrichment analysis of DNA5mC groups

**FIGURE 4:**
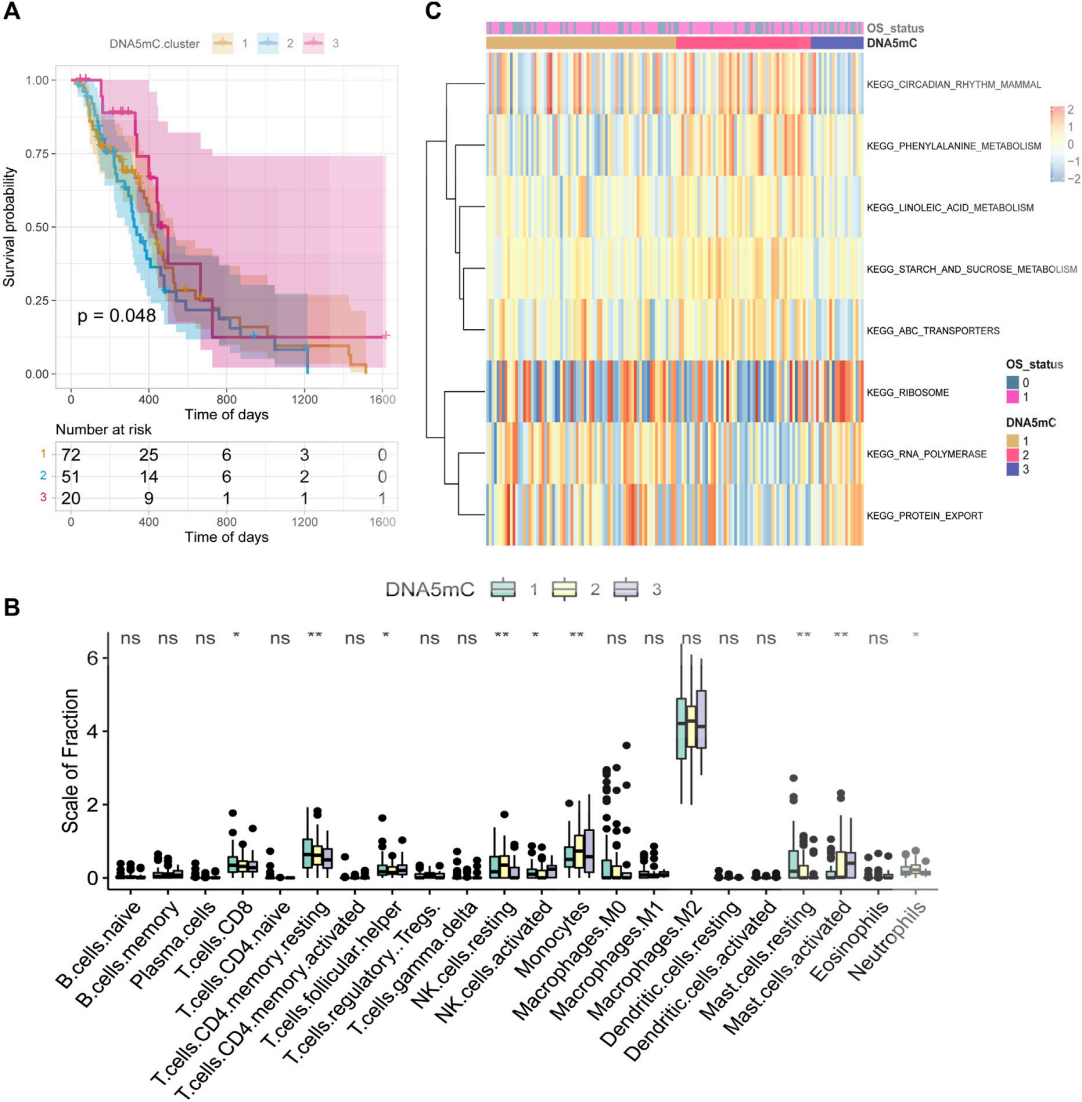
TCGA-GBM regulator identification and functional enrichment analysis. **(A)** Survival KM curve for tumor DNA5mC subtypes. **(B)** GBM DNA5mC subtypes differ in immune cell infiltration. **(C)** GSEA analysis of tumor DNA5mC groups.

Through unsupervised clustering analysis of 20 DNA5mC regulators, unique DNA5mC modification patterns were identified, which were then used to categorize patients for further investigation. A total of three DNA5mC subtypes were identified with distinct survival differences (Supplementary Figure S1). The median survival time for DNA5MC-3 was 659 days, which was mostly better than the median survival time for the other two subtypes. The median survival time of Dna5mc-2 was 437 days, which was highly associated with a poor prognosis (Figure 4A). A comparison was made between the immune cell infiltration of three DNA5mC subtypes (Figure 4B). We found that DNA5MC-1 and DNA5MC-2 subtypes were remarkably enriched in CD8 positive T cells, T cells CD4 memory resting, NK cells resting, and mast cells resting. DNA5mC-3 exhibited significantly high levels of infiltrated cells, including follicular helper cells, activated NK cells, and mast cells.

We also investigated the biological processes that differentiate DNA5mC subtypes using gene set enrichment (GSEA) analyses (Figure 4C). KEGG CIRCADIAN RHYTHM MAMMAL, KEGG PHENYLALANINE METABOLISM, and KEGG LINOLEIC ACID METABOLISM had the highest enrichment scores for DNA5mC-1 and DNA5mC-2 groups. There was a distinct increase in KEGG RIBOSOME activity, KEGG RNA polymerization activity, and KEGG protein export activity in the DNA5mC-3 subtype.

### Immune-related factor expression profile among DNA5mC subgroups

**FIGURE 5:**
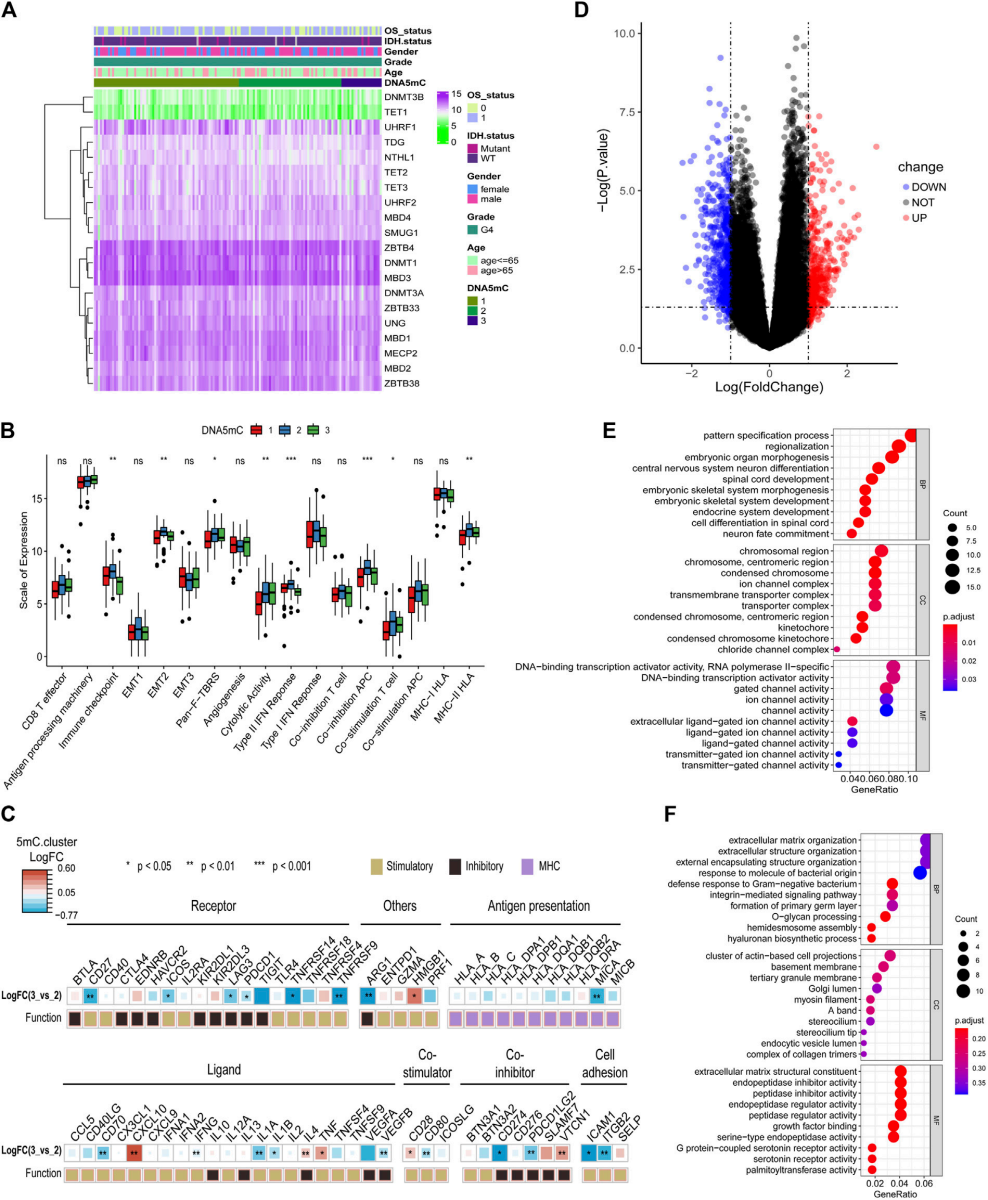
Tumor DNA5mC subtypes differ in the expression of immune-related factors. **(A)** Heatmap displaying the expression profiles of 20 DNA5mC genes. **(B)** Different immune-related factors subsets among three DNA5mC subsets. **(C)** A difference between DNA5mC-2 and -3 subtypes in immune-related factors. **(D)** Differential expression analysis between DNA5mC-2 and -3. **(E–F)** Down-regulated and up-regulated DEGs enriched with GO terms.

A relationship between DNA5mC subtypes and immune signaling factors in tumors is worth investigating, since immune-related signaling factors are crucial to the development of the tumor immune microenvironment. The expression profiles of DNA5mC-related genes among disctint subtypes and clinical features were observed (Figure 5A). We then found that several immune-related factors were various among DNA5mC subtypes (Figure 5B) In DNA5mC-1 and DNA5mC-2 subtypes, immune checkpoint, EMT2, Pan-F-TBRS, Type II IFN Response, Co-inhibition APC, Co-inhibition T cell, and MHC-II HLA have higher levels of activation signals, while Cytolytic Activity has higher levels in DNA5mC-3 subtype.

Differentially expressed genes (DEGs) among DNA5mC groups were analyzed to identify possible biological characteristics. A total pf 612 DEGs were found (Supplementary Table S3), including 234 that were up-regulated in DNA5mC-3, and 378 that were highly expressed in DNA5mC-2 group (Figure 5D). We found significant dysregulation of immune-related factors, including CD27, TNFRSF9, MICA, ICAM1, and ITGB2 (Figure 5C). GO functional enrichment analysis was also performed on DEGs, and the top 10 enriched pathways in each functional category were shown in Figures 5E,F. Many of the enriched pathways were associated with biological processes, including chromosome stability, nervous system development, and ion channels.

### Detection of DEG-related subtypes in glioblastoma multiforme

**FIGURE 6:**
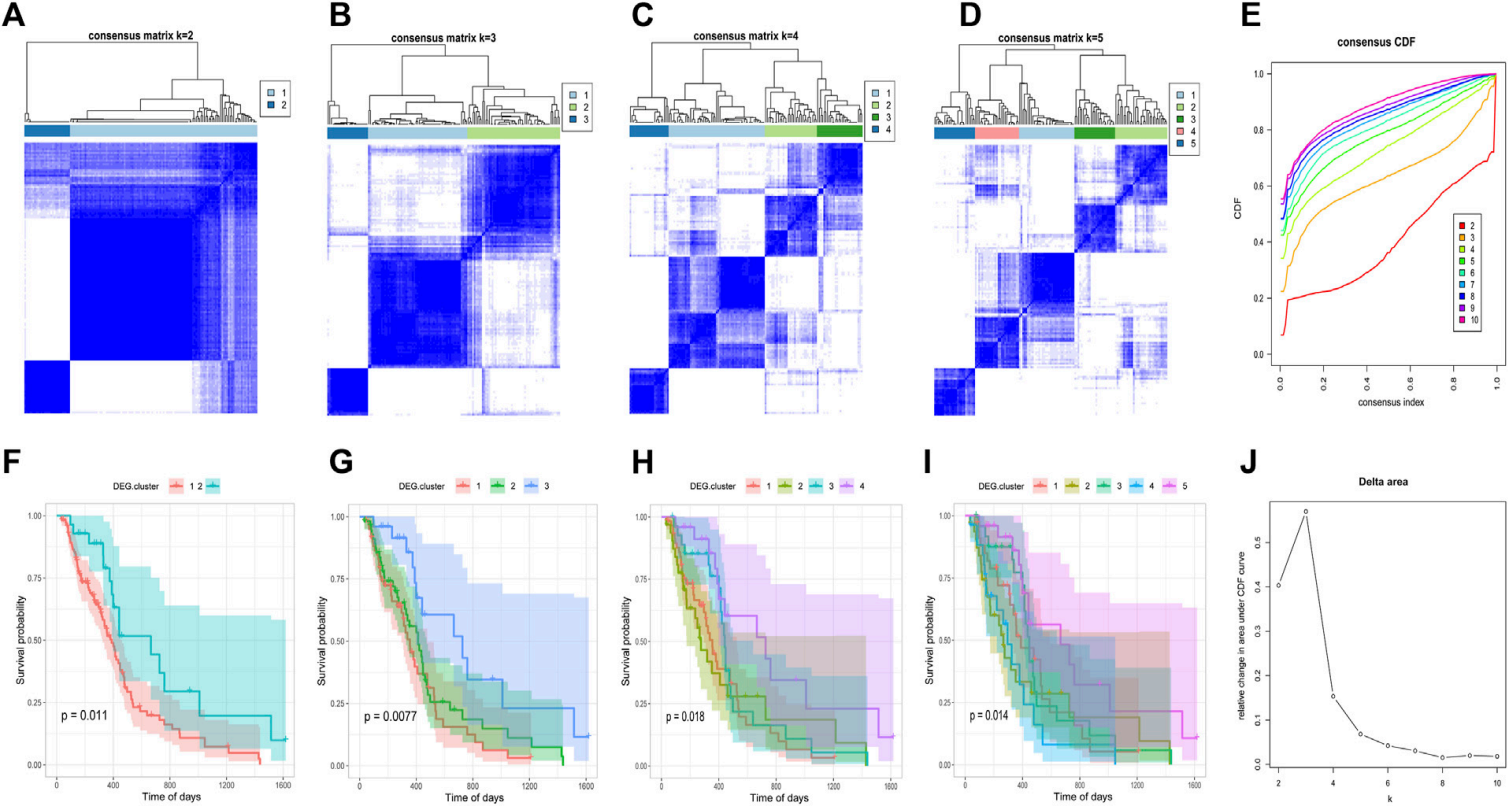
Consistent clustering of DEG. cluster-1 and -2 subgroups. **(A–D)** Sample clustering heat map when k = 2, k = 3, k = 4, k = 5, respectively. **(F–I)** Kaplan–Meier survival curves with k = 2, k = 3, k = 4, k = 5, respectively. **(E)** CDF curve distribution of consistency clustering. **(J)** Distribution of area under the CDF curve for consensus clustering.

**FIGURE 7:**
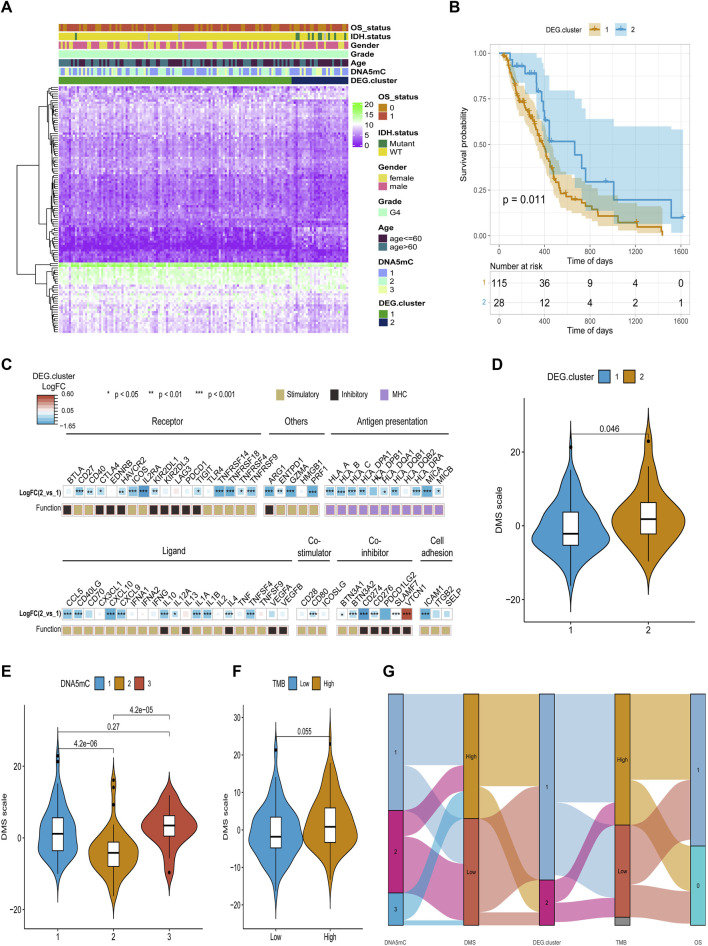
Identification and characterization of DEG. cluster-1 and -2 subtypes. **(A)** An overview of differential gene expression profiles. **(B)** Survival curves for distinct DEG. cluster groups. **(C)** A comparison of immune signaling factors among DEG. cluster subgroups. **(D–F)** Different DNA DMS for TMB groups, DNA5mC subtypes, and DEG. cluster subtypes. **(G)** Dynamic flow diagram of each GBM sample grouping and state transition.

After obtaining the 612 DNA5mC phenotype-related genes, we implemented unsupervised clustering analysis using DEG. cluster-1 and 2 (Figure 6) to categorize patients into various genomic subgroups. DEG signatures distinguished two distinct gene clusters in the analysis. IDH mutant status correlated primarily with DEG. cluster-2 (Figure 7A). A worse prognosis was associated with cluster-1 in GBM patients. Conversely, the prognosis of patients in DEG. cluster-2 was better (Figure 7B). Immune-related signaling factors were expressed differently in the two DNA5mC gene clusters (Figure 7C).

**FIGURE 8:**
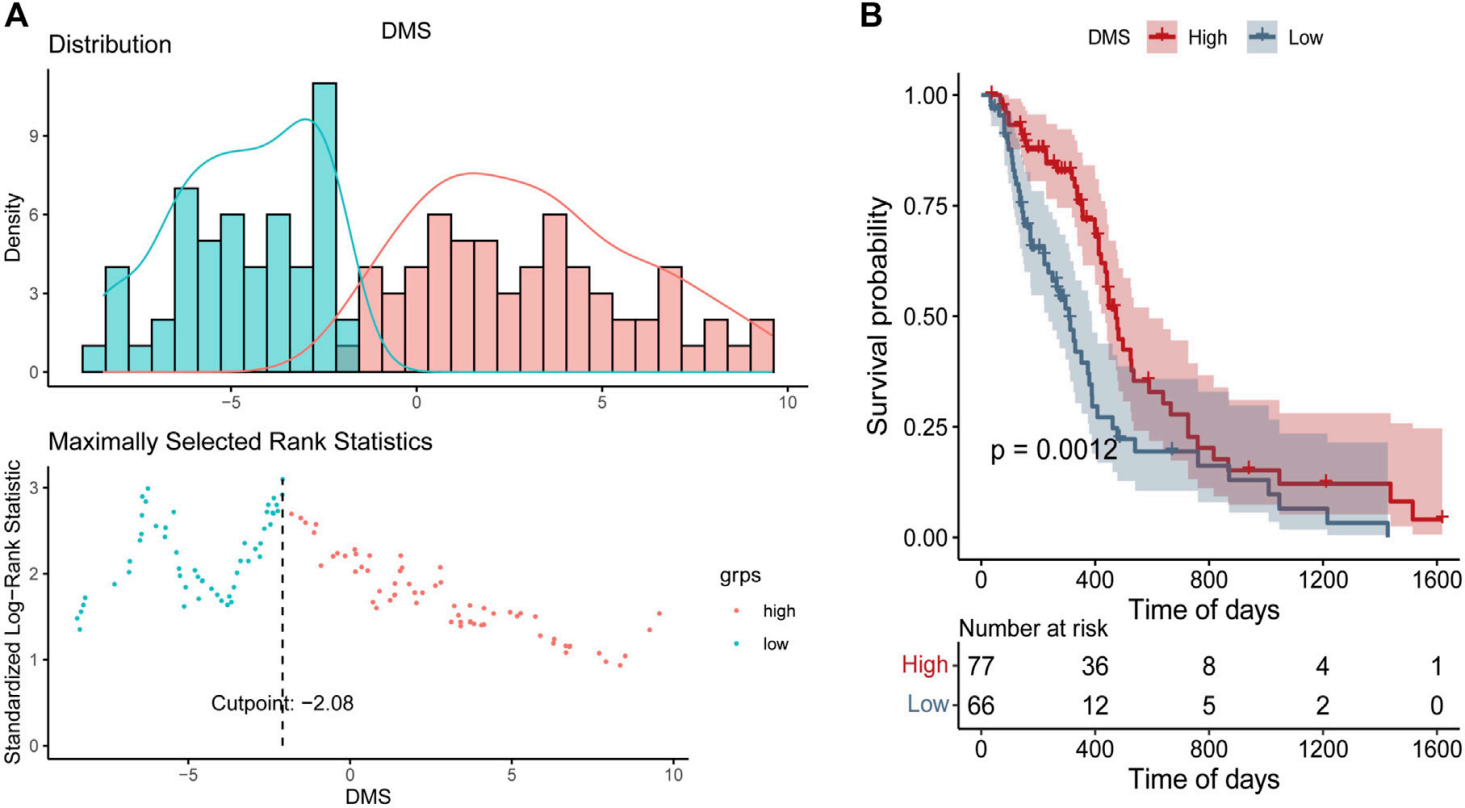
DNA Methylation Score (DMS) gradient grouping for tumors. **(A)** DMS distribution density statistics and optimal threshold determination. **(B)** Survival curves between groups with high and low DMS.

Based on DEGs between DNA5mC subtypes, dimensionality reduction analysis was performed on the DEG expression profiles using the PCA algorithm, and finally the weights of each sample in PC1 and PC2 were summed up to form DNA methylation score (DMS). Then, we calculated the optimal density gradient threshold (-2.08) of tumor DMS score to classify patients in TCGA-GBM dataset (Figure 8A). As shown in Figure 8B, the survival rates of the two groups with high and low DMS scores differed markedly. We further examined the distributions of DMS among tumor mutational burdens (TMBs), DNA5mC subtypes, and DEG. clusters, which indicated that significant differences existed between the above groups in terms of DMS. As shown in Figures 7D–G, high DMS score was enriched in high TMB group, DNA5mC-3 subgroups, and DEG. cluster-2 subgroups. Increasing evidence showed that anti-PD-1/PD-L1 immunotherapy produced a long-lasting therapeutic response in patients with high TMB status (Wang et al., 2020). These findings may offer novel perspectives on the mechanisms underlying gene mutation and DNA5mC status in GBM.

### Characterization of DNA methylation score in validation datasets

**FIGURE 9:**
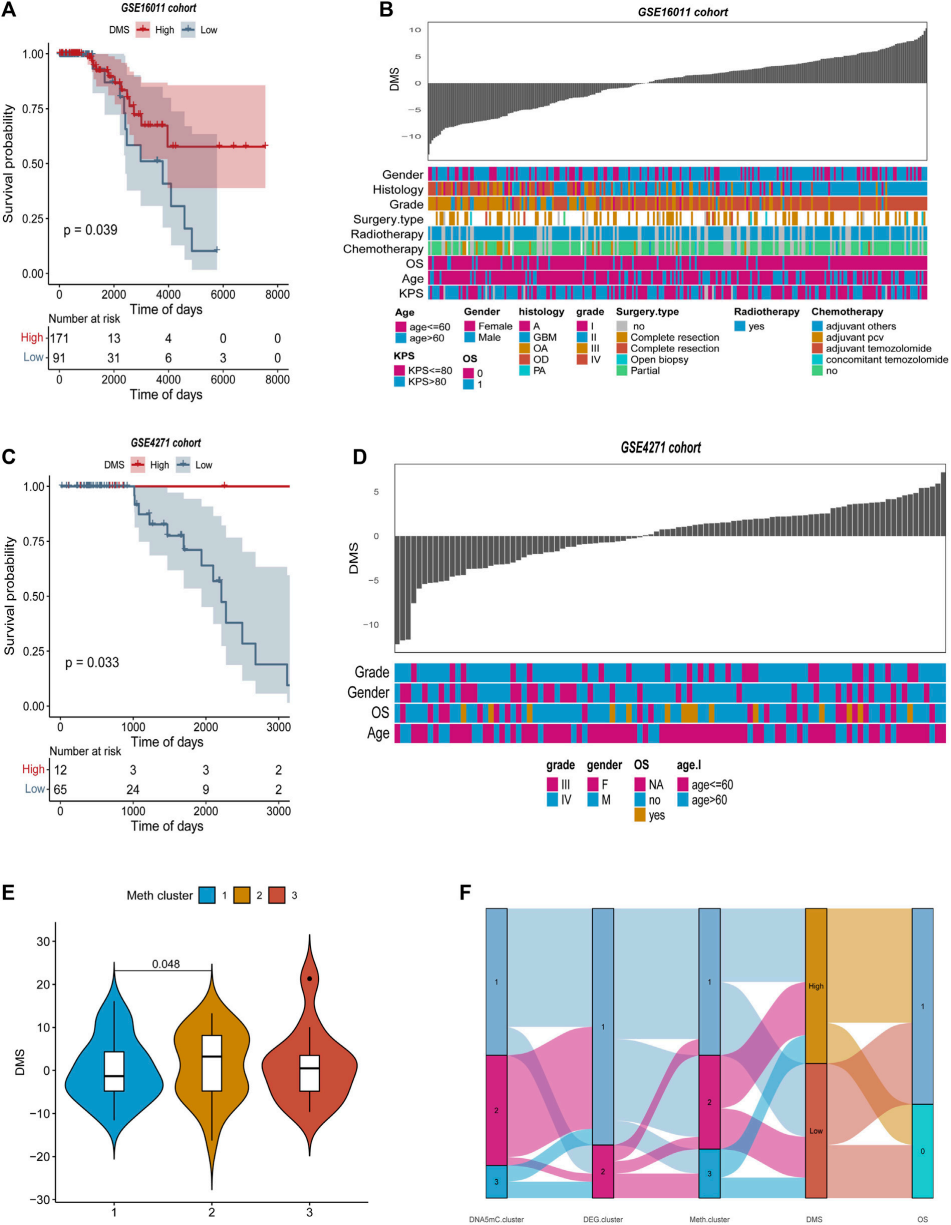
Features of tumor DNA methylation scores in external GEO cohorts. **(A–B)** Survival curves between high and low DMS groups in GSE16011 and GSE4271 datasets, separately. **(C–D)** Relationship between clinical special diagnosis and DMS distribution in GSE16011 and GSE4271 datasets, separately. **(E)** Different methylation subtypes differ in DMS. **(F)** A dynamic flow diagram illustrating each grouping and state transition of the tumor sample.

The DEGs-based DMS was further evaluated with two GEO datasets (GSE16011 and GSE4271) to further evaluate its robustness for predicting overall survival in GBM (Kassambara et al., 2017; Wu et al., 2021). The optimal density gradient threshold for tumor DMS and survival was calculated using the DEGs screened in the previous steps, and the ‘Survminer’ R package was used to calculate the DMS for GSE16011 and GSE4271 databases. The two GEO datasets were divided into two groups based on DMS scores, and an important difference in survival was observed between the low and high score groups (Figures 9A–C). DMS exhibited some correlation with other clinical characteristics in the two GEO datasets, as illustrated by the heatmap (Figures 9B–D). Further analysis showed that Meth. cluster-3 tended to have a lower DMS, which was associated with better prognosis. DMS in DNA5mC subtypes (Figures 9E,F) confirmed previous findings.

### Tumor DNA methylation score as a predictor of anti-PD-1/L1 immunotherapy outcome

**FIGURE 10:**
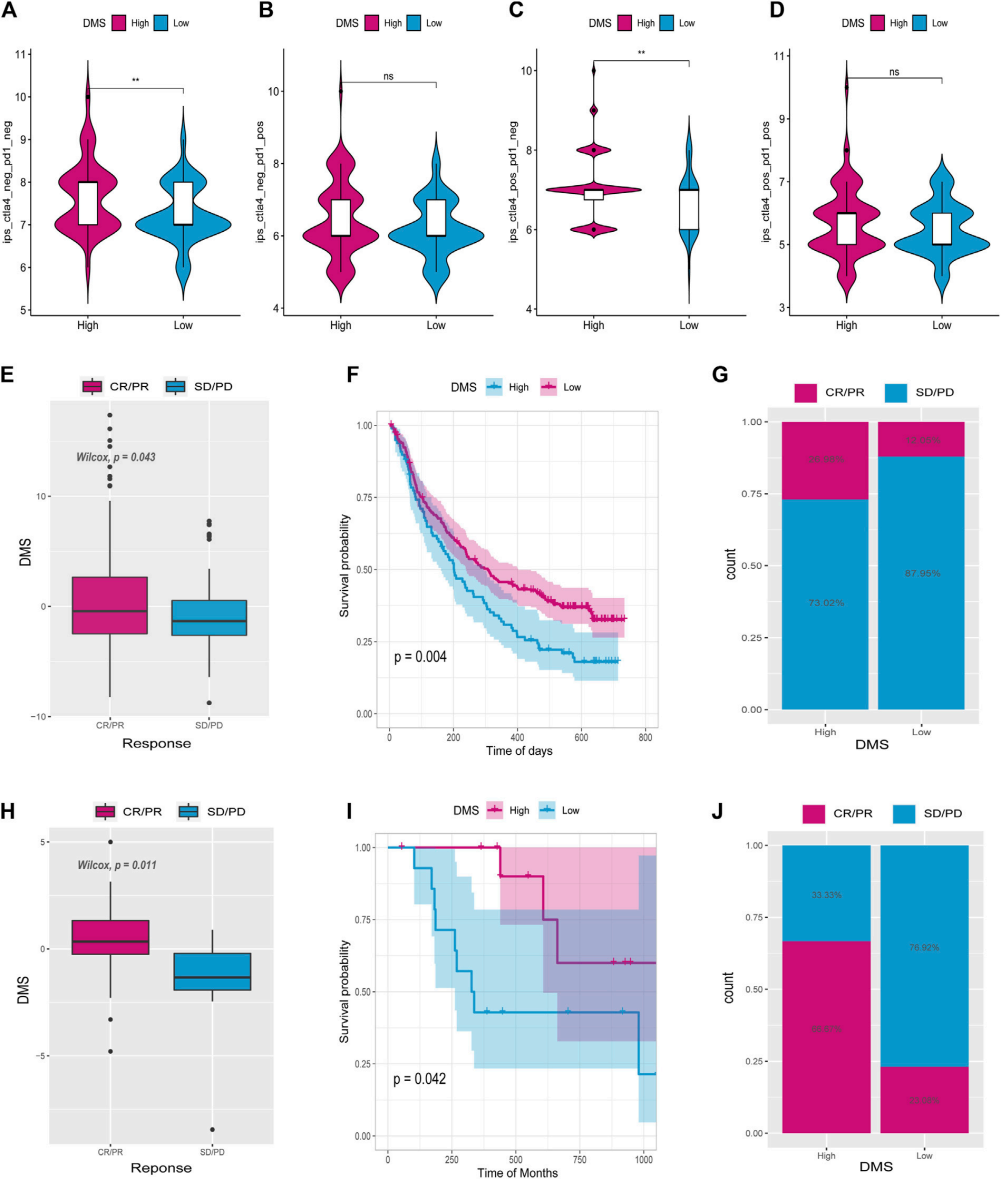
Tumor DNA methylation score (DMS) as a predictor of immunotherapeutic benefit. Immunophenoscore (IPS) differences between high and low DMS groups **(A–D)**. E and H show the difference in ICI scores between IMvigor210 and GSE78220 cohorts. **(F,I)** Survival curves for high and low ICI score groups in IMvigor210 and GSE78220 cohorts, respectively. **(G,J)** Differences between high and low ICI score groups in IMvigor210 and GSE78220 cohorts.

Immunophenoscore (IPS) assesses tumor immunogenicity and predicts how immunotherapy will treat various types of cancers (Charoentong et al., 2017). A significant difference was also observed between IPS scores in the high DNA methylation score (High_DMS) and the low DNA methylation score (Low_DMS) groups (Figures 10A–D). According to this finding, immunotherapy may be beneficial to patients with a high DMS.

There has been a significant progress in the treatment of cancer thanks to PD-L1 and PD-1 blockage immunotherapies (Hoffman-Censits et al., 2016). Using data from anti-PD-L1 (IMvigor210) and anti-PD-1 (GSE78220) cohorts, we studied whether the DMS signature could predict the effectiveness of immune checkpoint blockade therapy. It was found in both IMvigor210 (Figures 10E–G) and GSE78220 (Figures 10H–J) databases that individuals with high DMS showed considerable therapeutic benefits. Patients with high DMS responded well to anti-PD-1/L1 immunotherapy when compared to those with low DMS. According to these findings, DNA5mC-related DEGs may contribute to establishing a correlation between immunotherapy response and DNA methylation.

## Discussion

In glioma and neural development, DNA methylation and demethylation play critical roles in physiological and pathological processes (Papanicolau-Sengos and Aldape, 2022). Several DNA5mC regulators interact with DNA5mC modification to affect inflammation, innate immunity, and antitumor effect. There is a lack of understanding of the global TME infiltration features mediated *via* the integrated roles of multiple DNA5mC regulators. Thus, elucidating the role of DNA5mC modification patterns in TME cell infiltration will aid in the development of more effective immunotherapy strategies against TME.

In this study, we explored three DNA5mC methylation modification patterns based on 20 DNA5mC regulators. Infiltration of TME cells in these three patterns was prominently different. Furthermore, this study demonstrated changes in mRNA transcriptomes across different DNA5mC modification patterns significantly linked to DNA methylation and immune-related pathways. These differentially expressed genes were identified as DNA5mC-related signature genes. Then, two genomic subtypes were clustered based on DNA5mC signature genes, which were also correlated with immune activation. These findings revealed that the DNA5mC modification was of great significance in shaping different TME landscapes. Our understanding of infection by TME cells will be improved by analyzing DNA5mC modification patterns comprehensively.

A quantification of DNA5mC modification patterns has become imperative due to the individual heterogeneity of DNA5mC modifications. We developed a scoring system for evaluating DNA5mC modification patterns in GBM patients. DMS scores and tumor mutation burden showed a markedly correlation. Moreover, DMS scores were found to be reliable and robust tools for estimating TME infiltration patterns or tumor immune phenotypes from DNA5mC modification patterns of individual tumors. We also validated the predictive value of the DMS score in two cohorts that received anti-PD-1 and anti-PD-L immunotherapy. In addition, our study demonstrated that DNA5mC methylation patterns shaped different immune TME landscapes, suggesting DNA5mC modification may influence immune checkpoint blockade efficacy. PD-L1 expression, immune TME status, and mutations in DNA5mC genes could be more effective predictive biomarkers for immunotherapy than DNA5mC regulators alone.

To summarize, the DMS score could be used in clinical practice to assess the methylation patterns of DNA5mC as well as the corresponding TME cell infiltration within a GBM patient, to determine the immune phenotypes of tumors, and to guide clinical practices more effectively. The findings of our research provided new insights into improving immunotherapy patients’ clinical outcomes, discovering various tumor immune phenotypes, and advancing tailored cancer immunotherapy.

## Data Availability

The original contributions presented in the study are included in the article/Supplementary Material, further inquiries can be directed to the corresponding author.

## Supplementary material

SUPPLEMENTARY FIGURE S1 Consistent clustering of DNA5mC −1, −2, and −3 subgroups. (A-B)Sample clustering heat map when k=2, k=3, k=4, k=5, respectively. **(F-I)** Kaplan–Meier survival curves with k=2, k=3, k=4, k=5, respectively. **(E)** CDF curve distribution of consistency clustering. **(J)** Distribution of area under the CDF curve for consensus clustering.

SUPPLEMENTARY TABLE S1 TCGA-GBM clinical information statistics in the present study.

SUPPLEMENTARY TABLE S2 The overall infiltration results by CIBOERSORT algorithm.

SUPPLEMENTARY TABLE S3 Details of 612 DEGs among DNA5mC subtypes.
