# Supplementary figures and images for: DNA5mC Regulator-Mediated Molecular Clusters and Tumor Microenvironment Signatures in Glioblastoma
Source: Front Cell Dev Biol. 2022 Nov 8;10:1055567. doi: 10.3389/fcell.2022.1055567 (PMC9679379; doi:10.3389/fcell.2022.1055567)

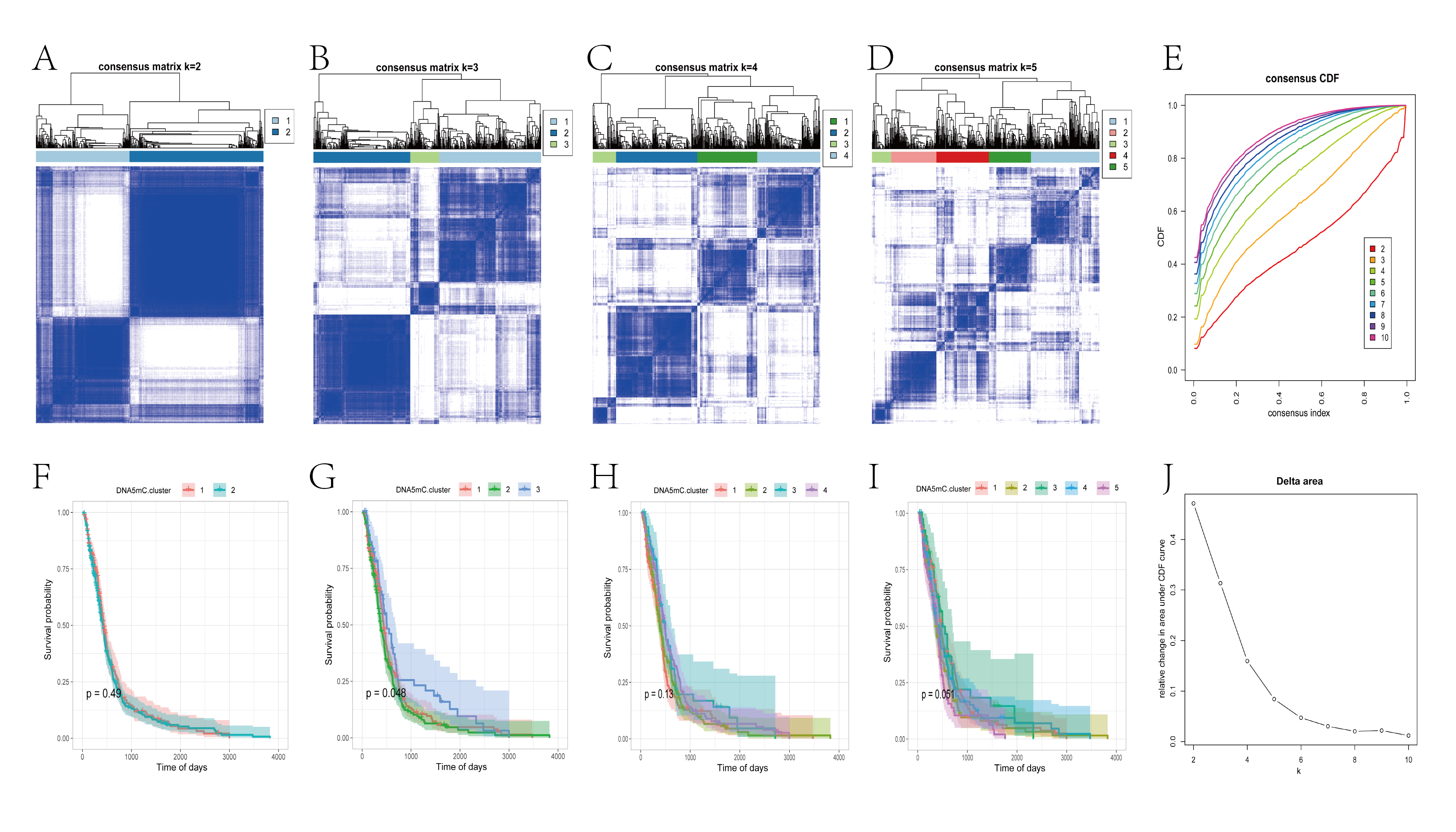

Supplement: Supplementary file 3 [file Image1.TIF]
